# Intratumoral ^198^Au Nanobrachytherapy Reduces Tumor Growth and Metastasis in a Murine Model of Triple-Negative Breast Cancer

**DOI:** 10.1021/acsomega.6c02355

**Published:** 2026-07-30

**Authors:** Mayara Santana Pinto, Camila de Almeida Salvego, Wilmmer Alexander A. Rosero, Paulo Victor dos Santos Tavares, Emerson Soares Bernardes, Orlando Rodrigues Junior, Pedro Zambianchi, Maria Elisa C. M. Rostelato, Martha Simões Ribeiro

**Affiliations:** † Center for Lasers and Applications, 119500 Nuclear and Energy Research Institute (IPEN-CNEN), São Paulo, São Paulo 05508-000, Brazil; ‡ Center of Radiation Technologies, 119500 Nuclear and Energy Research Institute (IPEN-CNEN), São Paulo, São Paulo 05508-000, Brazil; § Center for Ionizing Radiation Metrology, 119500 Nuclear and Energy Research Institute (IPEN-CNEN), São Paulo, São Paulo 05508-000, Brazil; ∥ Center of Radiopharmacy, 119500 Nuclear and Energy Research Institute (IPEN-CNEN), São Paulo, São Paulo 05508-000, Brazil; ⊥ 74354 Federal University of Technology - Paraná, Curitiba, Parana 80230-910, Brazil

## Abstract

Nanobrachytherapy (NB) uses radioactive nanoparticles (NPs) to deliver localized ionizing radiation within tumors, whereas photobiomodulation therapy (PBM) employs low-intensity light to modulate cellular responses. Here, we evaluated NB alone and combined with PBM using ^198^AuNPs and red laser irradiation in a murine triple-negative breast cancer (TNBC) model. Female mice were inoculated with 4T1 cells in the lower left mammary fat pad and, once tumors reached ∼0.1 cm^3^, were assigned to three groups: NB (intratumoral injection of ∼200 μCi ^198^AuNPs), PBM+NB (single red laser session before NB), and Control (PBS). Tumor progression, clinical signs, spleen mass, and lung metastases were evaluated over 23 days. Galectin-3 (Gal-3) expression was quantified by RT-qPCR, and biodistribution and dose delivery of ^198^AuNPs were assessed by gamma counting and Monte Carlo simulations. NB significantly reduced tumor growth, lung metastases, and splenomegaly and improved clinical status compared with controls, accompanied by decreased Gal-3 expression. Similar outcomes were observed with PBM+NB, with no additional therapeutic benefit compared with NB alone. Biodistribution confirmed preferential tumor retention of ^198^AuNPs, which declined over time. The estimated tumor dose rate was ∼9.34 mGy/s, decreasing to ∼4.14 mGy/s after 20 min, consistent with NP diffusion. Environmental dose estimates in the isolator remained below 34% of the tumor dose, supporting procedural safety with appropriate radiological protection. These findings demonstrate that ^198^Au-based NB effectively reduces tumor progression and metastatic burden in TNBC. Under the conditions tested, PBM did not modify therapeutic outcomes. The results support further investigation of localized radionuclide delivery using metallic NPs as an intratumoral radiation strategy.

## Graphical abstract

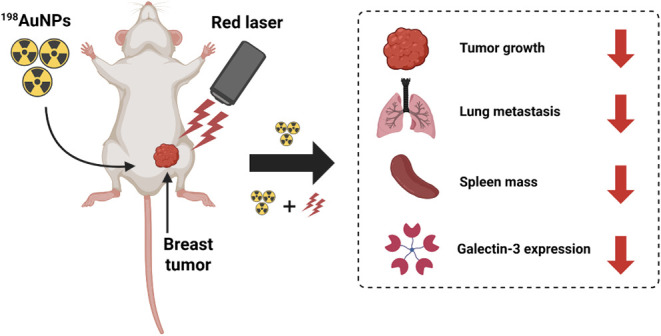

## Introduction

Cancer constitutes a significant global public health burden and remains a critical global health issue, standing as the foremost driver of mortality worldwide and responsible for nearly 10 million deaths in 2020.^1^ According to the World Health Organization (WHO), breast cancer (BC), together with lung, colorectal, liver, and stomach cancers, ranks among the leading causes of cancer incidence and mortality globally.

BC encompasses a heterogeneous group of neoplasms influenced by both genetic and environmental factors. Approximately 5–10% of BC cases are hereditary, with about 25% of these linked to germline mutations, particularly in the BRCA1 and BRCA2 genes.^2^ These mutations often give rise to more aggressive disease, and, together with molecular subtypes, contribute to the heterogeneity and clinical severity of BC.^3^

Triple-negative breast cancer (TNBC) is a highly aggressive molecular subtype, defined by the absence of estrogen and progesterone receptors (ER-, PR-) and the lack of HER2 overexpression, and is associated with an unfavorable prognosis.^4^ It can be further subdivided into six molecular categories, each with distinct clinical implications. This subclassification aids in tailoring treatment strategies, as TNBC exhibits considerable inter- and intratumoral heterogeneity.^5^ Standard therapy typically involves surgery when feasible, neoadjuvant chemotherapy, and, in selected cases, monoclonal antibody therapy. Additionally, external beam radiotherapy (RT) is generally recommended postmastectomy or after breast-conserving surgery in most patients.^5,6^

Recent advances have investigated the use of gold nanoparticles (AuNPs) as multifunctional tools in oncology combined with RT, including their use as radiosensitizers or photothermal enhancers to improve tumor control.^7,8^ Brachytherapy (BT) is an RT modality in which radioactive sources are placed directly within or adjacent to the tumor bed, enabling highly localized dose delivery while minimizing exposure to surrounding healthy tissues. BT is commonly employed in early stage BC for partial breast irradiation, particularly after lumpectomy, offering both precision and shorter treatment courses compared to conventional RT.^9^ In this scenario, nanobrachytherapy (NB) extends the concept of BT by using radioactive NPs to deliver localized, nanoscale radiation directly to the tumor, thereby reducing systemic toxicity and collateral tissue injury.^10^ Although NB has shown promising results in other malignancies, such as prostate cancer,^11,12^ no studies to date have investigated the application of ^198^AuNPs in TNBC, leaving its therapeutic potential in this context completely unexplored.

Additionally, light-based strategies, such as photobiomodulation therapy (PBM), have been explored as a radiomodifying agent in combination with RT.^13,14^ Evidence suggests that PBM may sensitize tumor cells to radiation while simultaneously protecting healthy cells from radiation-induced damage, potentially mitigating harmful side effects.^14^ The therapeutic efficacy of PBM depends on multiple parameters, including wavelength, fluence, fluence rate, and exposure time, and the optimal conditions for achieving consistent biological effects are still being defined.

In this context, the present study aimed to evaluate the therapeutic potential of NB with ^198^AuNPs in a preclinical TNBC model and to investigate whether PBM could modulate its effects. Using a murine TNBC model, we examined tumor growth, clinical signs, metastasis formation, and galectin-3 (Gal-3) expression as a marker of tumor progression following NB alone or in combination with PBM. In addition, the biodistribution of ^198^AuNPs was quantified by gamma counting^11^ and the radiation dose generated within the tumor was estimated using Monte Carlo simulations with the MCNP 6.3 code.^15^ Environmental radiation levels were assessed separately using TOPAS,^16^ in which alanine dosimeters positioned at different locations through the isolator were used to map the ambient dose field.

## Experimental Section

### Cell Line

Triple-negative murine mammary carcinoma cell line 4T1 (ATCC, CRL-2539) was selected for tumor induction. This lineage is described as a model of straightforward handling in both *in vitro* and *in vivo* studies.^17^ Cells previously stored in liquid nitrogen were thawed in RPMI 1640 medium (Sigma, USA) supplemented with 10% fetal bovine serum (Sigma, USA) and 1% antibiotic-antimycotic solution (Sigma, USA). Cells were maintained in an incubator (Sanyo, USA) at 37 °C and 5% CO_2_ for 2 days until reaching 70–80% confluency. At this point, 1.0 × 10^5^ cells were resuspended in 50 μL aliquots of cell medium and loaded into individual 1 mL hypodermic syringes.

### Tumor Model

This study was approved by the Ethics Committee on the Use of Animals of the Instituto de Pesquisas Energéticas e Nucleares (IPEN). Isogenic BALB/c female mice, approximately 6 weeks old and weighing around 20 g, were used. Animals were housed under 12-h light-dark cycles, with water and food provided *ad libitum*, and maintained in appropriate isolators for the entire experimental period.

Animals were anesthetized with 2.5% inhaled isoflurane, and the previously prepared cell aliquots were injected subcutaneously into the left lower mammary gland (third quadrant) after shaving and disinfection (Bonther, Brazil). After inoculation, animals were monitored daily to confirm tumor establishment by local palpation, assess clinical signs, and ensure overall welfare.

Sample size was estimated using the Experimental Design Assistant (https://eda.nc3rs.org.uk/eda/landing), based on the ability to detect a biologically relevant difference in tumor burden between groups. Given the well-established efficacy of NB approaches, an effect size corresponding to an approximate 60% reduction was assumed, with a standard deviation of 20%, a significance level of 0.05, and a statistical power of 90%. The EDA indicated a minimum of four animals per group. However, to account for potential attrition and interindividual variability, and to ensure robustness and reproducibility of the findings, six animals per group were included.

Once tumors reached 100 mm^3^ (14 days after cell inoculation),^14^ they were randomly assigned to three groups: (i) Control (N = 6; no treatment), (ii) NB (N = 6; inoculation with ^198^AuNPs only), and (iii) PBM+NB (N = 6; a single PBM exposure before ^198^AuNP inoculation). Handling, anesthesia, and procedural stress were equivalent across treated and control groups, ensuring appropriate benchmarks for intergroup comparisons. A standalone PBM group was not included due to ethical considerations in accordance with the 3Rs principle, particularly Reduction, which aims to minimize animal use. Additionally, previous work from our group using the 4T1 model demonstrated that a single PBM application, even across different fluences, does not produce a measurable antitumor effect.^18^ Therefore, the experimental design focused on evaluating the combined effect of PBM with NB.

### Treatments and Monitoring

Control animals received 30 μL of PBS (Sigma, USA), whereas animals in the NB and PBM+NB groups received a single intratumoral injection of 30 μL of ^198^AuNPs (corresponding to ∼1.4 × 10^14^ AuNPs per injection), delivering an activity of 200 μCi directly to the tumor site. AuNP concentration (∼4.7 × 10^15^ AuNP·mL^–1^) was estimated from the precursor concentration (0.03 M HAuCl_4_) by calculating the total number of gold atoms and dividing by the number of atoms per NP (∼3.8 × 10^3^ for 5 nm AuNPs). The activity of each preparation was measured using a dose calibrator (Capintec CRC-15W, USA), as it depends on neutron flux, irradiation time, and radioactive decay. During inoculation, anesthetized animals were individually positioned in an acrylic support system that maintained a safe distance from the operator.

^198^AuNPs coated with gum arabic (GA) were synthesized via neutron activation of metallic gold (^197^Au → ^198^Au) at the EA-R1 nuclear reactor (IPEN), followed by dissolution in aqua regia to generate an *in situ* radioactive chloroauric precursor and subsequent reduction with sodium citrate in the presence of GA as a stabilizing agent. The reaction was performed at 100 °C with vigorous stirring, resulting in rapid NP formation. Radioactive AuNPs can be produced by different neutron-based strategies, including (i) activation of preformed nanoparticles^19^ and (ii) irradiation-assisted synthesis via radiolytic reduction.^20^ In contrast, this study employed a radiochemical approach based on neutron activation of bulk metallic gold, followed by dissolution and NP synthesis from the radioactive precursor. As in previous reports, but distinct from classical methods,^21,22^ the precursor is generated *in situ*, avoiding commercial gold salts and minimizing handling steps. This strategy prevents irradiation-induced alterations to NP coatings and decouples radionuclide production from NP formation, enabling better control over synthesis while ensuring the intrinsic incorporation of ^198^Au.

Transmission electron microscopy (TEM) of nonradioactive samples showed spherical NPs with an average core size of ∼5 nm, while dynamic light scattering (DLS) indicated a hydrodynamic diameter of ∼50 nm due to aggregation in aqueous solution. The NPs exhibited good colloidal stability, confirmed by ζ-potential measurements and time-dependent DLS analyses, with no significant aggregation over time. Further details on NP synthesis and characterization have been reported elsewhere.^23^

Before ^198^AuNP administration, animals in the PBM+NB group received a single session of PBM with a red laser (MMOptics, Brazil). The irradiation parameters were 660 nm, 40 mW, 6 J, 150 J·cm^–2^, and 150 s, and a beam area of 0.04 cm^2^. The PBM regimen employed was selected based on previous works from our group demonstrating radiomodulatory effects under similar conditions.^14,24^ No visible skin damage was observed during treatment, and the applied parameters fall within the nonthermal range typically associated with PBM. Although local temperature was not directly measured, the low-power and exposure conditions used are not expected to cause significant thermal effects.

The application was performed at a single point, with the laser tip in direct contact and positioned at a 90° angle to the center of the tumor surface. Tumor growth was measured every 3 days using a digital caliper, and tumor volume was calculated according to the equation:^14^ ${\mathrm{V(cm}}^{3}{)=0.5\;x\;\mathrm{length}\;x\;\mathrm{width}}^{2}$

Animal clinical status, including fur appearance (piloerection), posture, movement, and respiratory alterations, along with body mass, were monitored weekly (Figure 1). Each animal was monitored individually for 120 s. The individual scores for each clinical sign were summed to generate a total score per animal, and group totals were then calculated. Higher scores reflected a more compromised clinical condition, whereas lower scores indicated better overall health.

**Figure 1:**
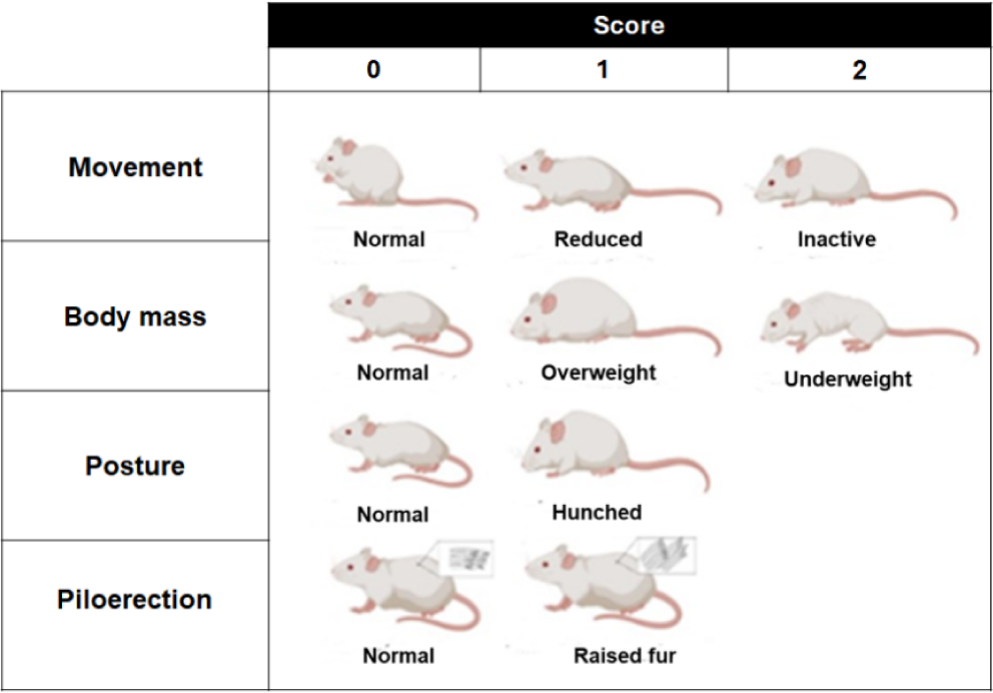
Clinical scoring criteria used for animal health assessment. Created with BioRender.

Euthanasia was performed on day 23 using an overdose of anesthetics (60 mg/kg xylazine and 235 mg/kg ketamine), in accordance with institutional ethical guidelines. Based on a previous study, animals in this TNBC model reached predefined humane end points at approximately this time point due to tumor burden and welfare criteria, and were therefore euthanized to minimize suffering.^25^ Consequently, survival analysis was not performed. Lungs, spleens, and tumors were collected and fixed in 10% buffered formaldehyde for 24 h, then stored in 70% ethanol for an additional 24 h prior to further analysis. Lungs were examined for macroscopic metastatic nodules, and spleens were evaluated by mass.

### Gal-3 Gene Expression Assessment

For real-time quantitative polymerase chain reaction (RT-qPCR) analysis, tumor tissues were excised and homogenized in TRIzol reagent (Sigma, USA) for total RNA extraction, according to the manufacturer’s instructions. RNA concentration and purity were assessed spectrophotometrically (260/280 nm ratio). Complementary DNA (cDNA) was synthesized from 1 μg of total RNA using the high-capacity cDNA reverse transcription kit (Applied Biosystems, USA), following the manufacturer’s protocol.

RT-qPCR was performed in triplicate using Power SYBR Green Master Mix (Applied Biosystems, USA) on a real-time PCR system (Applied Biosystems, USA). Amplification conditions consisted of an initial denaturation step followed by 40 cycles of denaturation and annealing/extension (as specified by the manufacturer).

Relative gene expression was calculated using the comparative ΔΔCt method, with β-actin as the endogenous control and normalization to the control group, as previously described.^26^ Table 1 presents the forward and reverse primer sequences used for β-actin and Gal-3 amplification.^27^

**Table 1:** Forward and Reverse Primer Sequences Used for the Amplification of β-Actin and Gal-3 by RT-qPCR

Primer	Forward 5′–3′	Reverse 5′–3′
β-Actin	CTAAGGCCAACCGTGAAAAG	ACCAGAGGCATACAGGGACA
Gal-3	GGTGGAGCACTAATCAGGAAA	CGGATATCCTTGAGGGTTTG

### Biodistribution Assay

For the radioactive NP biodistribution analysis, the animals were euthanized at 3 h, and 23 days after ^198^AuNP inoculation. The excised organs were immediately weighed, and their radioactivity was quantified using a gamma counter (2470 Automatic Gamma Counter, PerkinElmer, USA). The activity detected in each organ (liver, spleen, lungs, muscle, kidneys, intestine, stomach, tumor, blood, and others) was expressed as the percentage of the injected dose per gram of tissue (%ID/g).

### Estimated Therapeutic Dose Delivered to the Tumor

The radiation dose deposited in the tumor and adjacent organs was determined through computational simulations performed with the Monte Carlo N-Particle Transport Code, version 6.3 (MCNP 6.3), a well-established and widely validated platform for dosimetric calculations.^28–30^ All simulations were carried out on a workstation equipped with an Intel Core i9-13900K processor (24 cores, 32 threads, 5.8 GHz). In this modeling approach, the mouse body, and the tumor were approximated by a set of ellipsoidal volumes. The major and minor axes assigned to each ellipsoid (cm) were: mouse 5.8 × 2.5, lungs 1.4 × 0.7, liver 2.3 × 0.9, kidneys 0.9 × 0.6, bladder 0.6 × 0.5, and a tumor defined as 0.73 × 0.56 (Figure 2A). Each structure was assigned elemental compositions consistent with standard human tissue references.^31^ Upon injection, the 30 μL of the solution containing ^198^AuNPs was modeled by a spherical droplet of initial radius 0.1928 cm, which was completely filled with a hexagonal lattice of AuNPs, by using the MCNP 6.3 LAT = 2 card. To account for the subsequent 3-dimensional diffusion of ^198^AuNPs within the tumor, the average distance traveled by the NPs was calculated using the root-mean-square distance (RMSD) expression $\sqrt{\left\langle r^{2}\right\rangle }=\sqrt{6Dt}$ , where D is the diffusion coefficient and t is the elapsed time. The expression of D is given by the classical Stokes–Einstein diffusion coefficient D = k_B_T/6πηa, where k_B_ is the Boltzmann constant, t is the absolute temperature, η is the dynamic viscosity, and a is the nanoparticle radius.^32^ In this paper, T = 37 °C, η = 8.9 × 10^–6^ cm^2^/s^33^ and a = 2.5 nm. The present model also assumes that diffusion is approximately isotropic and that the droplet will remain spherical while the NPs remain within the tumor, implying a maximum diffusion time of about 20 min.

**Figure 2:**
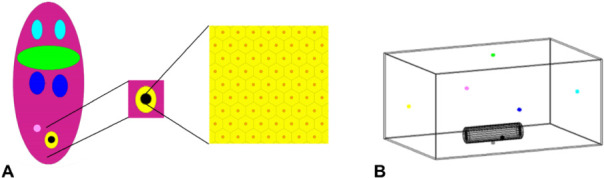
Schematic representations of the geometries used in Monte Carlo simulations for delivered dose (A) and ambient dose mapping (B). In (A), ellipses illustrate the mouse body (pink), lungs (cyan), liver (green), kidneys (blue), bladder (purple), and tumor (yellow). The black sphere represents the intratumoral ^198^AuNP droplet, and the zoom displays its honeycomb-like lattice structure used in the simulation. In (B), alanine dosimeters (green, yellow, cyan, blue, purple, and gray pellets) were distributed throughout the isolator to evaluate ambient dose. The black cylinder represents the BALB/c mouse, and the black sphere corresponds to the tumor containing ^198^AuNPs in the simulation model.

### Environmental Dose Estimation

The dose in the acrylic isolator environment was estimated using TOPAS (TOol for PArticle Simulations), version 3.8,^16,34^ to characterize the energy deposition around animals treated with ^198^AuNPs during the experimental period. The simulation geometry reproduced the experimental acrylic isolator (285 × 251 × 240 mm; Alesco, Brazil), modeled as a rectangular enclosure with poly­(methyl methacrylate) (PMMA) walls. Inside the chamber, a cylindrical water-equivalent phantom representing a BALB/c mouse (87 mm length × 25 mm width × 20 mm height) was positioned on the floor of the isolator, in accordance with the experimental setup (Figure 2B).

A tumor region was defined within the phantom at the anatomical location corresponding to the inoculation area and was considered the volume of the radioactive source. The ^198^AuNPs were assumed to be homogeneously distributed throughout this tumor region. To represent stochastic radioactive decay, individual emission events were randomly sampled at positions within the source volume, according to the decay characteristics of ^198^Au, including the beta emission spectrum reported by the International Atomic Energy Agency (IAEA) radionuclide database.^35^

Six virtual alanine dosimeters, with dimensions corresponding to the alanine pellet (4.8 mm in diameter and 2.99 mm in thickness), were positioned at representative locations throughout the isolator (top, lateral walls, center, and floor level), centered along the height and width of its rectangular faces, to assess the spatial distribution of the absorbed dose in the surrounding environment (Figure 2B). The alanine/EPR dosimeter is a secondary standard for absorbed doses in the dose range of 1 Gy to 150 kGy and for photon and electron energies between 0.1 and 30 MeV.^36^ Additionally, virtual alanine dosimeters were adopted to reproduce an experimentally feasible and clinically established environmental monitoring approach, thereby enhancing the translational relevance of the simulation model.

The physical models followed the standard TOPAS/Geant4 configuration (https://www.topasmc.org/). The electromagnetic interactions of photons and electrons were modeled using the G4EmStandard, which includes the photoelectric effect, Compton scattering, pair production, ionization, multiple scattering, and bremsstrahlung processes.^16,34^ The absorbed dose was calculated using the volumetric parameter DoseToMedium, which represents the average energy deposited per unit mass of the local medium, and the results were expressed as a percentage of dose.

### Statistical Analysis

Results are presented as means ± standard deviation (SD). Data normality was assessed using the Shapiro-Wilk test to guide the choice between parametric and nonparametric analyses. For normally distributed data, comparisons were performed using ANOVA followed by Tukey’s post hoc test. For non-normally distributed data, the Kruskal–Wallis test followed by Mann–Whitney post hoc comparisons was applied.^37^ Statistical significance was defined as *p* < 0.05. All analyses were conducted in OriginPro 2018.

## Results and Discussion

Because tumor growth followed an exponential pattern, it was modeled as V­(t) = V_0_·exp­(kt), where V_0_ is the initial volume and k is the growth constant. Taking the natural logarithm of both sides gives ln­[V­(t)] = ln­(V_0_) + kt, so k corresponds to the slope of the resulting linear plot (Figure 3A). The growth constant of the control group was significantly higher than that of both the NB and PBM+NB groups (0.12 ± 0.01 day^–1^ vs 0.06 ± 0.01 day^–1^ vs 0.09 ± 0.01 day^–1^, respectively). Moreover, the NB group exhibited significantly slower tumor growth than the PBM+NB group.

**Figure 3:**
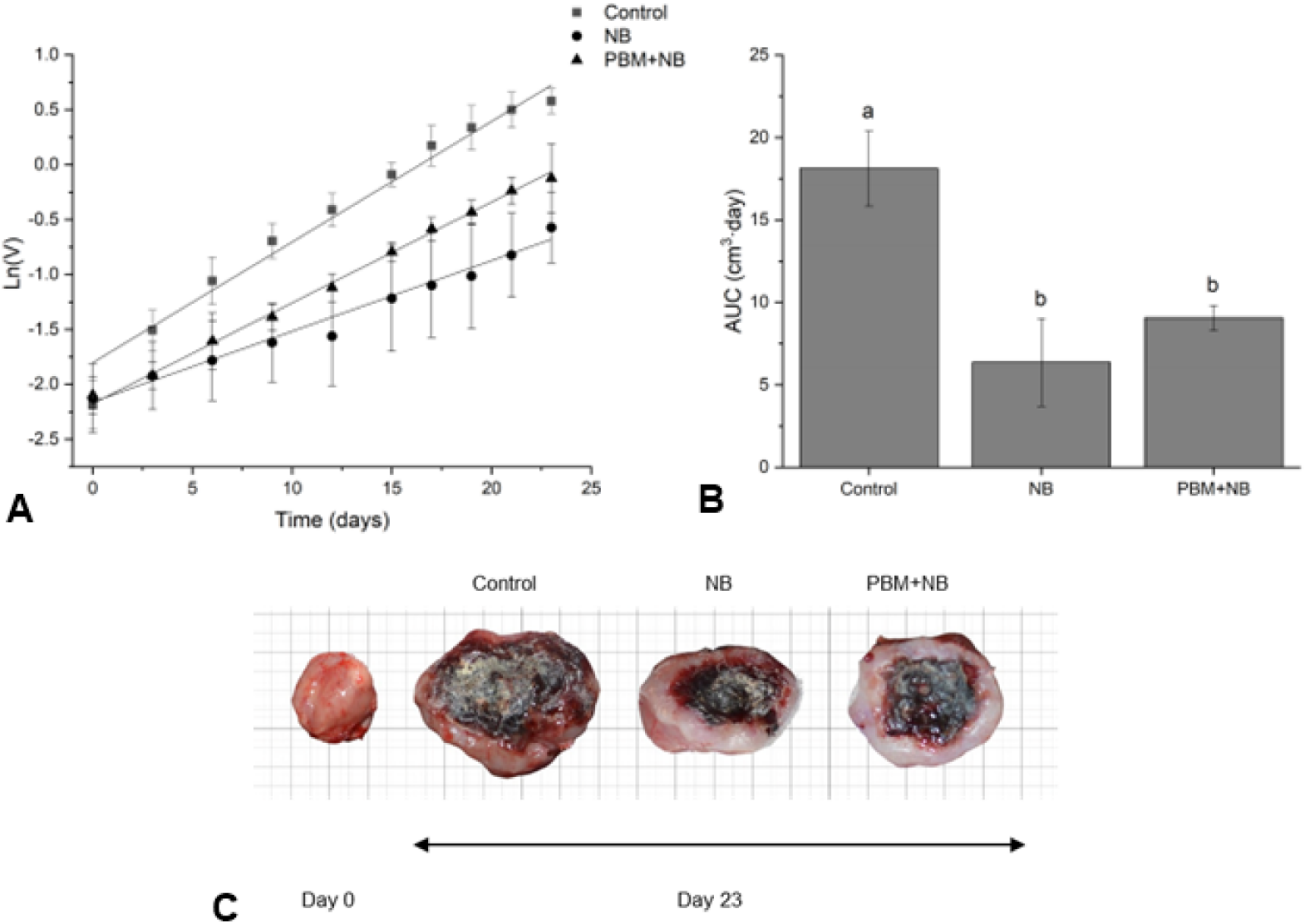
A) Linearized tumor growth curves over time for the Control, NB, and PBM+NB groups. The curve slopes differed statistically (*p* < 0.05). B) Mean values ± SD of the area under the tumor growth curves, representing overall tumor burden throughout the experimental period. Bars with different letters (a, b) denote statistically significant differences between groups (*p* < 0.05). C) Representative tumors collected on day 23 from each group, illustrating growth inhibition in the NB and PBM+NB treatments. For comparison, an image of the tumor at baseline (day 0) is also shown.

We also calculated the area under the tumor growth curves (AUC) (Figure 3B). The AUC reflects the overall tumor burden during the experimental period, representing cumulative growth over time. Interestingly, the NB and PBM+NB groups showed similar growth profiles (6.4 ± 2.6 cm^3^·day vs 9.1 ± 0.75 cm^3^·day), whereas the control group exhibited a significantly greater tumor burden than both treated groups (18.1 ± 2.3 cm^3^·day). Representative tumor images from day 23 are shown in Figure 3C.

Although both NB and NB+PBM significantly reduced tumor growth compared to the control group, complete tumor regression was not observed at later stages of tumor development. This outcome is likely related to the highly aggressive nature of the 4T1 model, in which tumors progress rapidly and become less responsive once established.^38^ In this context, slowing tumor progression and reducing tumor burden represent meaningful therapeutic effects. Moreover, NB delivers continuous low-dose radiation, which is expected to modulate tumor progression rather than induce rapid tumor shrinkage, particularly in aggressive tumors.

Figure 4 illustrates the clinical progression of the animals throughout the experiment. During the first week, no measurable clinical signs were detected, although 70% of the animals began to show small necrotic spots. By week 2, mild piloerection became apparent. From week 3 onward, animals in the control group exhibited a noticeable decline in clinical conditions, including persistent piloerection and a moderate reduction in mobility. Early in that same week, some animals also showed reduced movement of the left hind limb, corresponding to the tumor site. In the final week, piloerection was evident across all groups, and 75% of the animals displayed postural alterations. From week 3, the NB and PBM+NB groups showed significantly lower clinical scores, indicating better overall condition compared with controls.

**Figure 4:**
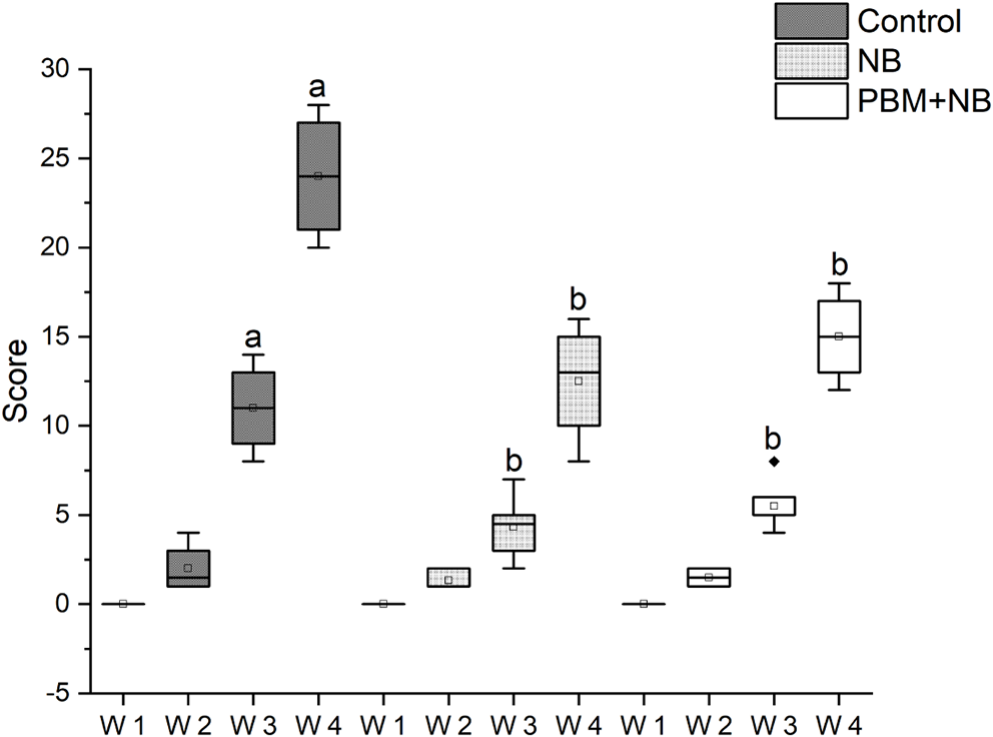
Box plot showing the score variation among groups (Control, NB, and PBM+NB) over 4 weeks (W1–W4). Data are presented as median, interquartile range, and minimum-maximum values (N = 6). Clinical signs were scored individually for each animal, and group scores reflect the sum of these individual values. During the first week, macroscopic clinical signs were absent or negligible in all animals. Different letters denote statistically significant differences between groups (*p* < 0.05).

Our findings show that both NB and PBM+NB slowed neoplasm progression, evidenced by reduced tumor growth over time, and improved clinical scores compared with the control group. These results align with previous reports indicating that radioactive NPs can inhibit tumor development in preclinical models.^23,39^ PBM was combined with NB to investigate whether the two modalities could act synergistically, thereby enhancing therapeutic efficacy and improving treatment outcomes.

In cancer therapy, neoadjuvant treatments are administered before the primary intervention to reduce tumor burden and facilitate resection, whereas adjuvant treatments are given afterward to eliminate residual microscopic disease, reduce recurrence, and enhance overall therapeutic effectiveness. Although supportive therapies do not always mitigate adverse effects, some approaches, including PBM, have been explored for their potential to modulate treatment-related toxicity.^40^ However, administering a PBM session before NB did not yield additional therapeutic benefits compared with NB alone.

Previous studies from our group have demonstrated that PBM can act as a radiomodifier in TNBC models.^14,24^ In our study, similar PBM parameters were applied. However, NB alone was sufficient to reduce tumor burden, and no additional benefit was observed with PBM. We also conducted studies using alternative protocols, including a two-session PBM regimen, with the second application performed 3 days after the first, corresponding to the half-life of ^198^Au, as well as a sequence in which NB was applied prior to PBM. Neither approach resulted in improved outcomes.^25^ Since PBM acts primarily by modulating mitochondrial activity and redox signaling, the persistent radiation-induced damage and oxidative stress associated with NB may have limited its effectiveness under these conditions. We hypothesize that the local dose delivered by the ^198^AuNPs, primarily via low-LET β-emissions, was likely sufficient to overshadow any biological modulation introduced by PBM. In practice, beta particles emitted from ^198^Au deposit their energy within a short-range around the NPs, meaning that radiation alone accounted for most of the therapeutic effect, leaving little measurable opportunity for PBM to further enhance the response. Nevertheless, different PBM irradiation parameters, including variations in wavelength, fluence, timing, and treatment schedule, may yield distinct outcomes and should be explored in future studies.

*Post-mortem* analyses on day 23 focused on detecting abnormalities such as splenomegaly and metastatic dissemination, both characteristic of this tumor lineage.^38^ Lung nodules were observed in all groups. However, the control group showed a markedly higher metastatic burden, with approximately 22 metastatic foci, whereas the NB and PBM+NB groups displayed significantly fewer foci, with approximately 14 and 15 nodules, respectively (Figure 5A and C). The treated animals also exhibited significantly lower mean spleen masses (approximately 772.7 ± 146.5 mg and 716.3 ± 137.7 mg for NB and PBM+NB, respectively) compared to the control group (1168.5 ± 120.7 mg) at the end of the experiment (Figure 5B).

**Figure 5:**
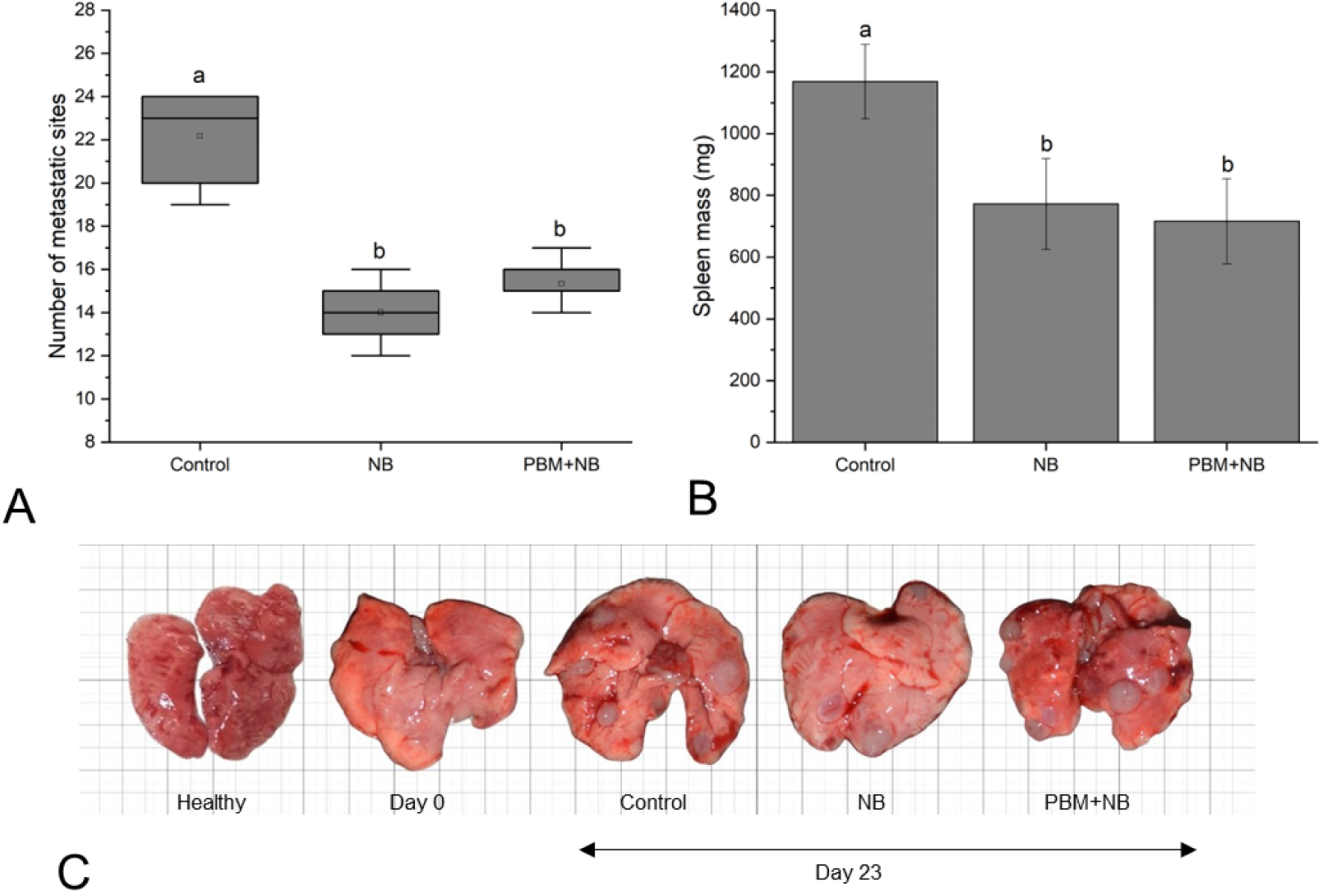
A) Box plot showing the number of metastatic nodules. Data are presented as median, interquartile range, and minimum-maximum values. B) Mean values ± SD of the spleen mass of animals from each experimental group on day 23. Different letters denote statistically significant differences between groups (*p* < 0.05). C) Representative lung images from the Control, NB, and PBM+NB groups on day 23. For comparison, images of healthy lungs and baseline lungs (day 0) are also included.

Although treatments did not completely prevent pulmonary metastasis or splenomegaly, treated animals exhibited fewer metastatic lung nodules and reduced spleen mass. Splenomegaly is common in 4T1-bearing mice and is associated with platelet sequestration, impaired splenic function, and disruptions in the immune microenvironment,^41^ all of which correlate with poorer cancer prognosis.^42^ Additionally, lung metastasis is a hallmark of the 4T1 TNBC model, which closely mimics the aggressive metastatic behavior of human stage IV breast cancer, typically showing early dissemination to the lungs within two to 3 weeks after tumor implantation.^38^

Intratumoral RT, although spatially restricted, can elicit systemic antitumor effects that extend beyond the treated lesion. Local tumor irradiation promotes immunogenic cell death, releasing tumor-associated antigens, cytokines, and danger-associated molecular patterns that remodel the tumor microenvironment and enhance antigen presentation. This process can ultimately drive the activation and expansion of tumor-specific T cells that can traffic systemically and target distant lesions. This phenomenon is classically described as the abscopal effect, in which regression of nonirradiated tumors occurs following localized RT.^43^ Additionally, intratumoral RT has been proposed as a form of *in situ* vaccination, whereby locally primed tumor-specific T cells mediate responses at distant metastatic sites.^44^ Other studies further demonstrate that localized RT can convert focal tumor damage into systemic antitumor immunity through coordinated immune activation processes, reinforcing the concept of radiation-induced immune priming.^45–47^ This framework is particularly relevant in the 4T1 model, which is characterized by aggressive growth, early dissemination, and spontaneous metastasis, closely resembling advanced human TNBC.^48^ In this model, local RT rarely leads to regression of advanced tumors but may still influence metastatic progression.^14,24^ In this context, the impact of intratumoral RT in metastatic disease reflects the interplay between local tumor control and systemic immune responses. Importantly, in aggressive metastatic models, effective management of the primary tumor remains clinically relevant, as it may delay disease progression and preserve overall condition. Here, NB improved local tumor control, improved clinical status, and reduced lung metastases, with no additional benefit observed with PBM.

In breast cancer, the expression and prognostic significance of Gal-3 remain controversial, with systematic reviews reporting conflicting associations with metastatic disease and clinical outcomes.^49,50^ Nevertheless, Gal-3 has been described as a molecular indicator of tumor behavior, with higher expression often associated with increased invasiveness, metastatic potential, and remodeling of the tumor microenvironment.^51^ We observed significantly higher Gal-3 levels in the control group (about 72%) than in the NB and PBM+NB groups (about 53%), respectively. Although the NB group had lower Gal-3 expression compared to the PBM+NB group, no statistically significant differences were detected (Figure 6A).

**Figure 6:**
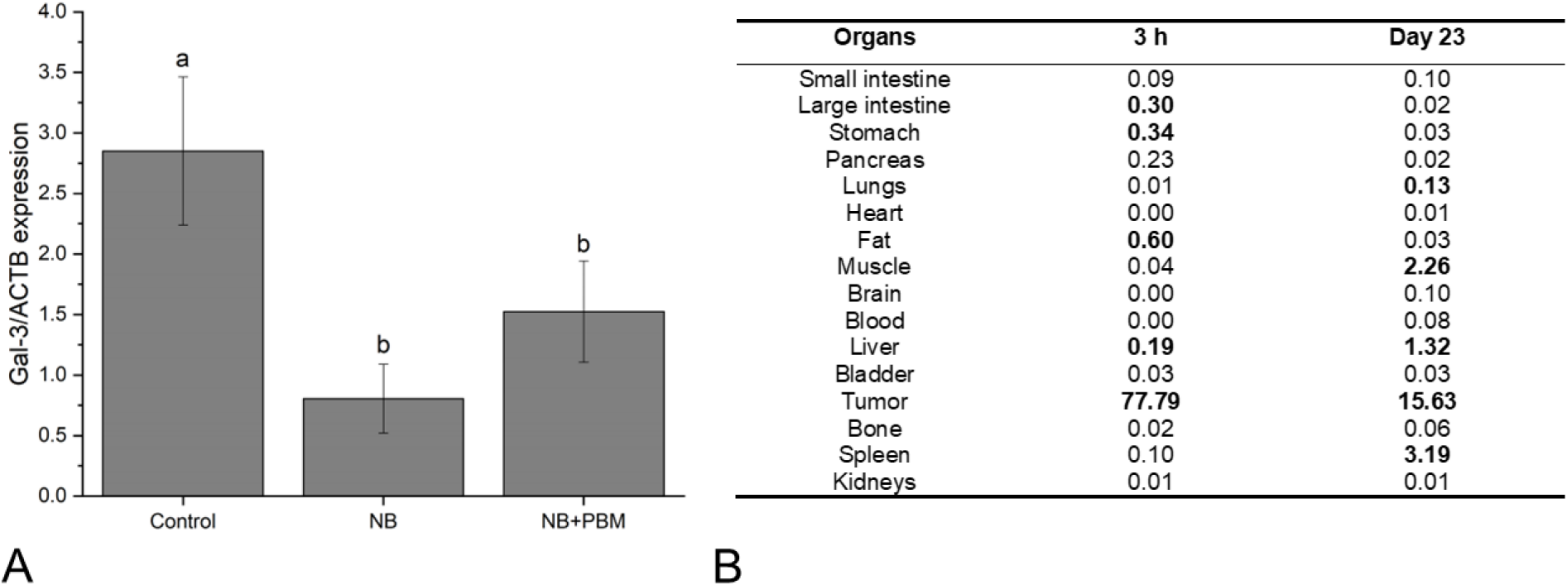
A) Mean values ± SD of Gal-3/ACTB expression levels in Control, NB, and NB+PBM groups. Bars with different letters denote statistically significant differences (*p* < 0.05). B) Tissue biodistribution of ^198^AuNPs, showing organ-specific accumulation patterns expressed as %ID/g.

NB and NB+PBM groups showed reduced Gal-3 expression, a result that agrees with previous reports linking lower Gal-3 levels to more favorable tumor behavior.^27^ Across different cancer types, Gal-3 has been associated with increased proliferation, angiogenesis, survival, and metastatic potential, including in BC.^51^ It has also been associated with clinicopathological features such as lymph node metastasis, estrogen receptor expression, and patient age, although its prognostic value remains limited and context-dependent, with no overall association with survival. Instead, its clinical relevance appears to depend on cellular localization, with cytoplasmic expression associated with better outcomes and combined cytoplasmic and nuclear localization linked to worse prognosis.^50^ In our study, the control group exhibited the highest Gal-3 expression, consistent with its greater metastatic burden and sustained tumor growth. Gal-3 often rises in stressful tumor microenvironments, particularly in hypoxic regions where ROS-driven signaling is elevated, and this may help explain the increased levels observed in untreated tumors. The reduction after NB likely reflects the direct cytotoxic effects of ^198^AuNPs, which decrease tumor cell viability and may dampen ROS-related pathways that drive Gal-3 upregulation. We assume that these radiation-induced changes are sufficient to suppress Gal-3 expression in this setting.

Biodistribution studies have become essential for determining the accumulation of AuNPs in target tissues and their partial or complete elimination.^52^ The biodistribution analysis quantified total radioactivity and did not distinguish between intact NPs, degradation products, or free ^198^Au. However, the platform is expected to remain stable under physiological conditions,^23^ suggesting that the detected signal predominantly reflects particle-associated activity. Importantly, the purpose of this analysis was to evaluate overall localization and retention of radioactivity, which remains informative regardless of its specific physicochemical form.

The highest concentration of ^198^Au was detected in the target mammary region at both 3 h and 23 days after NP inoculation (Figure 6B). However, by day 23, NP uptake in the tumor region had decreased by approximately 5-fold compared with the initial accumulation. Radioactive gold was also found in the large intestine, stomach, pancreas, fat, liver, and to a lesser extent in the small intestine, lungs, muscle, bone, bladder, spleen, and kidneys at 3 h after ^198^AuNP administration, likely reflecting limited systemic transport through blood and lymphatic circulation. These values gradually declined over time, and a clearer biodistribution pattern emerged at euthanasia, particularly in the spleen and liver, which is consistent with their central roles in processing and clearing foreign materials.^53^ The predominant retention in the tumor can be explained by the enhanced permeability and retention (EPR) effect, through which NPs accumulate in tumor microenvironments owing to abnormal vascular permeability and impaired lymphatic drainage.^54^ The clearance pattern identified here aligns well with previously reported data.^55,56^

Beyond these general biodistribution patterns, NP morphology is also a key determinant of tumor uptake and intratumoral distribution. For example, Black et al. demonstrated that ^198^Au-doped spherical NPs exhibited higher tumor accumulation, whereas anisotropic structures such as nanorods and nanocages showed deeper intratumoral penetration and more homogeneous distribution throughout the tumor mass.^57^ These findings highlight an important trade-off: spherical NPs tend to favor tumor uptake due to more efficient circulation and EPR-mediated accumulation, while anisotropic nanostructures may enhance penetration into tumor cores. From an NB perspective, this distinction is highly relevant, as dose distribution depends not only on total tumor uptake but also on spatial distribution within the tumor. In our study, we employed spherical ^198^AuNPs, which are widely used in NB due to their reproducible synthesis, colloidal stability, and predictable biodistribution. While anisotropic NPs may offer advantages in intratumoral penetration, spherical NPs provide a more controlled and consistent platform, which is critical for dosimetric reliability and reproducibility in localized radiation delivery.

Additionally, the choice of GA as a stabilizing agent further supports this approach. As a natural polysaccharide, GA provides excellent biocompatibility and intrinsic colloidal stabilization, reducing aggregation without requiring complex surface functionalization,^10,21,22^ Compared with PEGylation,^58^ which confers a “stealth effect” by reducing protein adsorption and immune recognition and thereby prolonging circulation time, GA may favor enhanced tumor retention. A comparison between GA and bovine serum albumin (BSA) coatings highlights important differences. BSA-coated ^198^AuNPs can promote biological interactions and potential targeting, but may be more susceptible to radiation-induced structural changes.^11^ In contrast, GA provides greater colloidal stability and resistance to aggregation, maintaining NP integrity even under radioactive conditions.^23^ Although targeted systems (e.g., integrin αvβ3-functionalized NPs) can enhance specificity, they increase formulation complexity and variability.^19,59^ Overall, GA-stabilized spherical ^198^AuNPs provide a robust and reproducible platform that combines stability, biocompatibility, and suitable tumor retention for NB applications.

The results of the MCNP6.3 simulations, including the spatial distribution of radiation emitted by the injected ^198^AuNPs at the moment of inoculation and 20 min later (when they begin to diffuse beyond the tumor), as well as the dose rate delivered to the tumor and surrounding tissues, are presented in Figure 7.

**Figure 7:**
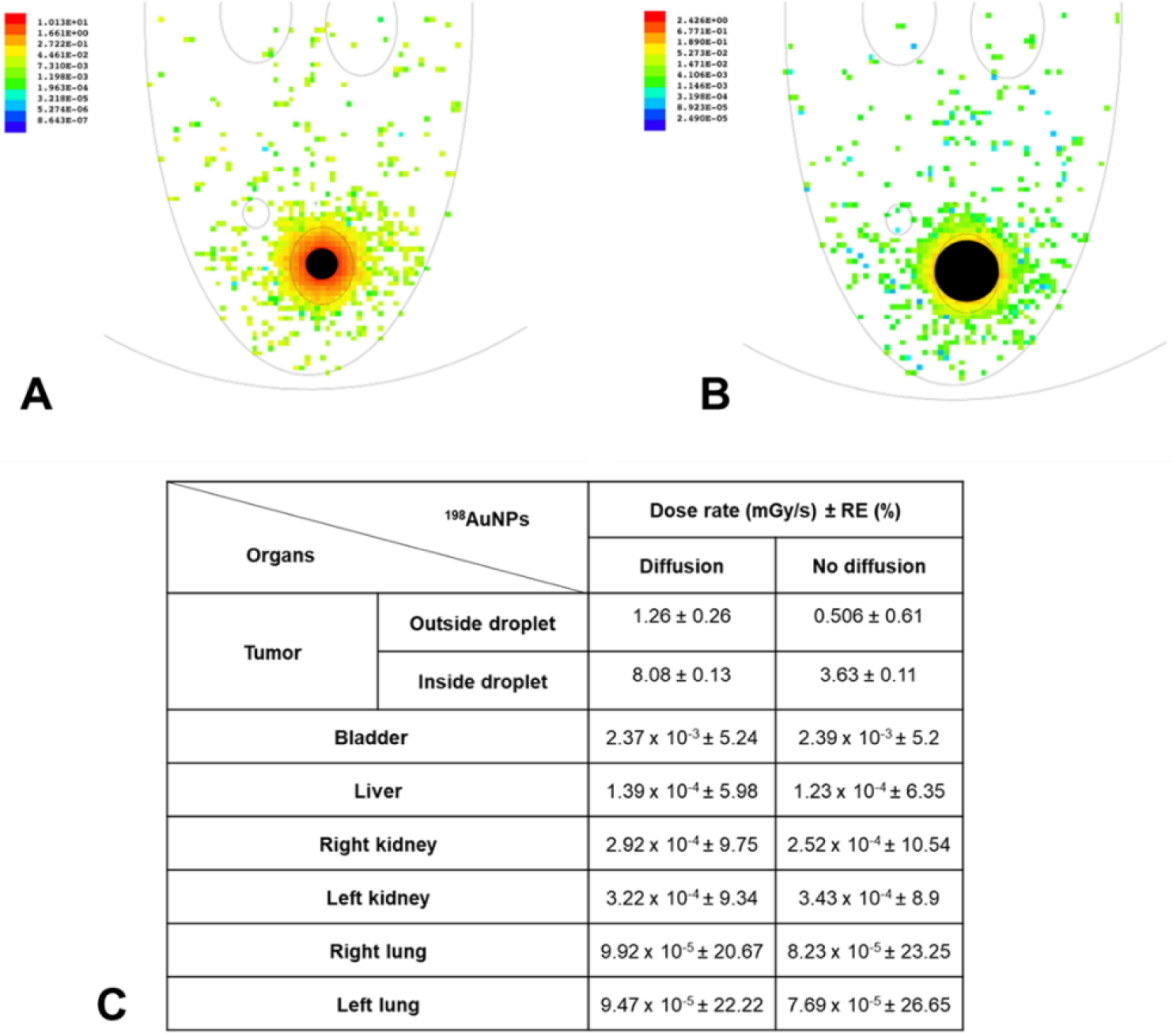
Spatial dose-rate distributions generated by ^198^AuNPs in the tumor region. (A) Simulation performed at time zero, when ^198^AuNPs remain confined to the intratumoral droplet, resulting in a highly concentrated dose around the injection site. (B) Simulation after 20 min, when ^198^AuNPs have diffused beyond the tumor borders, producing a broader and more dispersed dose pattern throughout the surrounding tissue. Color intensity represents dose rate per voxel, with warmer colors indicating higher values. (C) Mean dose rate (mGy/s ± relative error, RE) calculated for the tumor and surrounding organs at time zero and 20 min after ^198^AuNP administration.

Figure 7A exhibits the dose-rate distribution corresponding to the moment immediately after intratumoral injection, when the ^198^AuNPs were still confined to the droplet. Under this condition, energy deposition remained sharply localized, with the highest dose centered around the injection site and a steep attenuation with distance. After 20 min, the ^198^AuNPs migrate away from the original droplet, reducing the local dose peak and generating a dose field that becomes less narrowly centered on the original source region (Figure 7B). The estimated initial dose rate in the tumor was approximately 9.34 ± 0.29% mGy/s, decreasing by about 44% after 20 min (4.14 ± 0.62% mGy/s). In contrast, the dose rates in nearby organs remained essentially unchanged after diffusion, within the relative uncertainties, ranging from ∼2.4 ± 5.2% μGy/s in the bladder to ∼0.77 ± 26.65% μGy/s in the left lung (Figure 7C).

For the environmental dose mapping, particle trajectories were simulated for an initial set of emissions (Figure 8). The magenta trajectories represent the short-range, highly directional paths of β^–^ emissions originating from discrete decay points within the NP cluster (Figure 8A). In contrast to beta particles, the magenta trajectories display long-range, penetrating paths of γ rays that propagate throughout the entire simulated chamber (Figure 8B).

**Figure 8:**
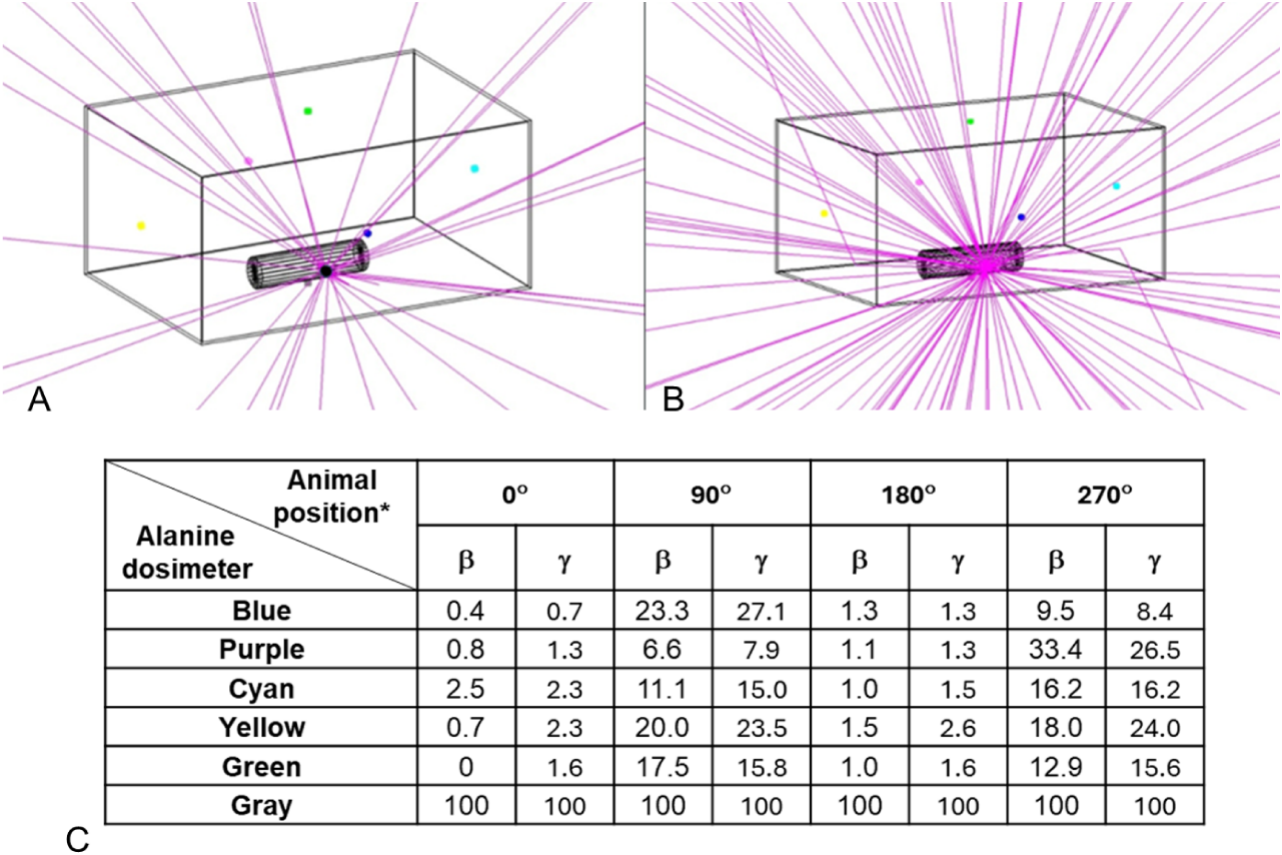
Spatial distributions of beta-particle (A) and gamma-photon trajectories (B) originating from the tumor containing ^198^AuNPs, represented by the black sphere inside the BALB/c mouse model (cylinder), used for environmental dose mapping. Colored pellets represent alanine dosimeters positioned at different locations throughout the isolator to estimate ambient dose. C) Percentage dose distribution associated with β^–^ and γ emissions. *Animal position relative to each alanine dosimeter is shown clockwise.

The spatial distribution patterns obtained from the alanine dosimeters showed that the highest signal was recorded in the detector positioned directly beneath the cylindrical mouse phantom, with progressively lower values as the detectors were placed farther from the tumor region (Figure 8C). As expected, both emission types (β^–^ and γ) were influenced by animal orientation and detector position, producing distinct gradients around the source. The spatial course (a steep decline in radiation intensity with increasing distance from the NP-containing tumor) suggests that emissions from the ^198^AuNPs remain largely confined to the immediate vicinity of the animal. This indicates that the use of radioactive NPs does not appreciably alter ambient dose levels in the laboratory. Taken together, these findings support the safe handling of NB in preclinical settings and suggest that, with proper radiological evaluation, similar strategies may be adapted in future stages of clinical development.

## Conclusion

Altogether, our findings demonstrate that NB using ^198^AuNPs effectively reduced tumor growth in a preclinical TNBC model and decreased Gal-3 levels, without evidence of worsened clinical condition or increased metastatic burden. The lack of additional benefit from PBM before NB suggests that the therapeutic response is predominantly driven by β^–^ radiation. Biodistribution of ^198^AuNPs confirmed their predominant retention at the inoculation site, consistent with Monte Carlo simulations indicating that the highest dose rates were confined to the tumor, with only minimal contribution to surrounding organs. Environmental dose mapping showed that radiation levels within the isolator remained below 34% of those measured in the tumor, supporting the safe execution of the procedure under appropriate radiological protection. Collectively, these results highlight the therapeutic potential of ^198^AuNP-based NB for localized treatment of TNBC and provide a quantitative basis for further refinement of this strategy.
